# Research advances and future perspectives of nanomaterials for depression therapy: a scientometric review

**DOI:** 10.1186/s12951-026-04226-3

**Published:** 2026-02-27

**Authors:** Dong Xu, Chao Guo, Wang-Ting Li, Yi Ding, Kai Gao, Wei Zhang, Mei-Na Zhao, Xing-Ru Tao, Jing-Wen Wang

**Affiliations:** Department of Pharmacy, Xijing Hospital, The Fourth Military Medical University, 127 Changle West Street, Xi’an, 710032 China

**Keywords:** Nanomaterials, Depression, Scientometrics, Diagnosis, Nano drug delivery system

## Abstract

**Background:**

Depressive disorder is one of the most common mental health conditions with significant repercussions on both the physical and psychological health of affected individuals. In recent years, an increasing number of studies have been undertaken to evaluate nanomaterials for the diagnosis and treatment of depression. This study aims to objectively and comprehensively summarize the research advances and future perspectives of nanomaterials for depression therapy via scientometric analysis.

**Methods:**

Literature related to nanomaterials for depression therapy was retrieved from Web of Science Core Collection, Scopus, and PubMed databases. Scientometric analysis and visualization were primarily performed using Bibliometrix and included publications, research topics, countries, institutions, journals, high-frequency keyword analysis, and keyword cluster analysis. Based on the results of this scientometric analysis, the article presents a detailed discussion and summary of the potential of nanomaterials for the diagnosis and treatment of depression.

**Results:**

A total of 343 articles related to the applications of nanomaterials in the treatment of depression were included, with an increasing number of publications noted each year. These studies focused primarily on three areas: materials science, nanotechnology, and pharmacy/chemistry. China, India, and Iran are the top three countries in this field. The most influential institutions are the Egyptian Knowledge Bank, Cairo University, Chinese Academy of Sciences, and Tehran University of Medical Sciences. The top three journals are *Microchimica Acta*, *International Journal of Pharmaceutics*, and *Journal of Nanoscience and Nanotechnology*. The results of keyword analysis revealed that the main areas of interest are diagnosis, nanomaterials with antidepressant activity, nano-drug delivery systems, and nanotoxicity.

**Conclusion:**

Based on the results of the scientometric analysis, this study discusses the diagnosis, treatment, and current limitations of nanomaterials for depression therapy. This review will inspire novel ideas for the development of nanomaterials with applications in depression therapy.

**Graphical Abstract:**

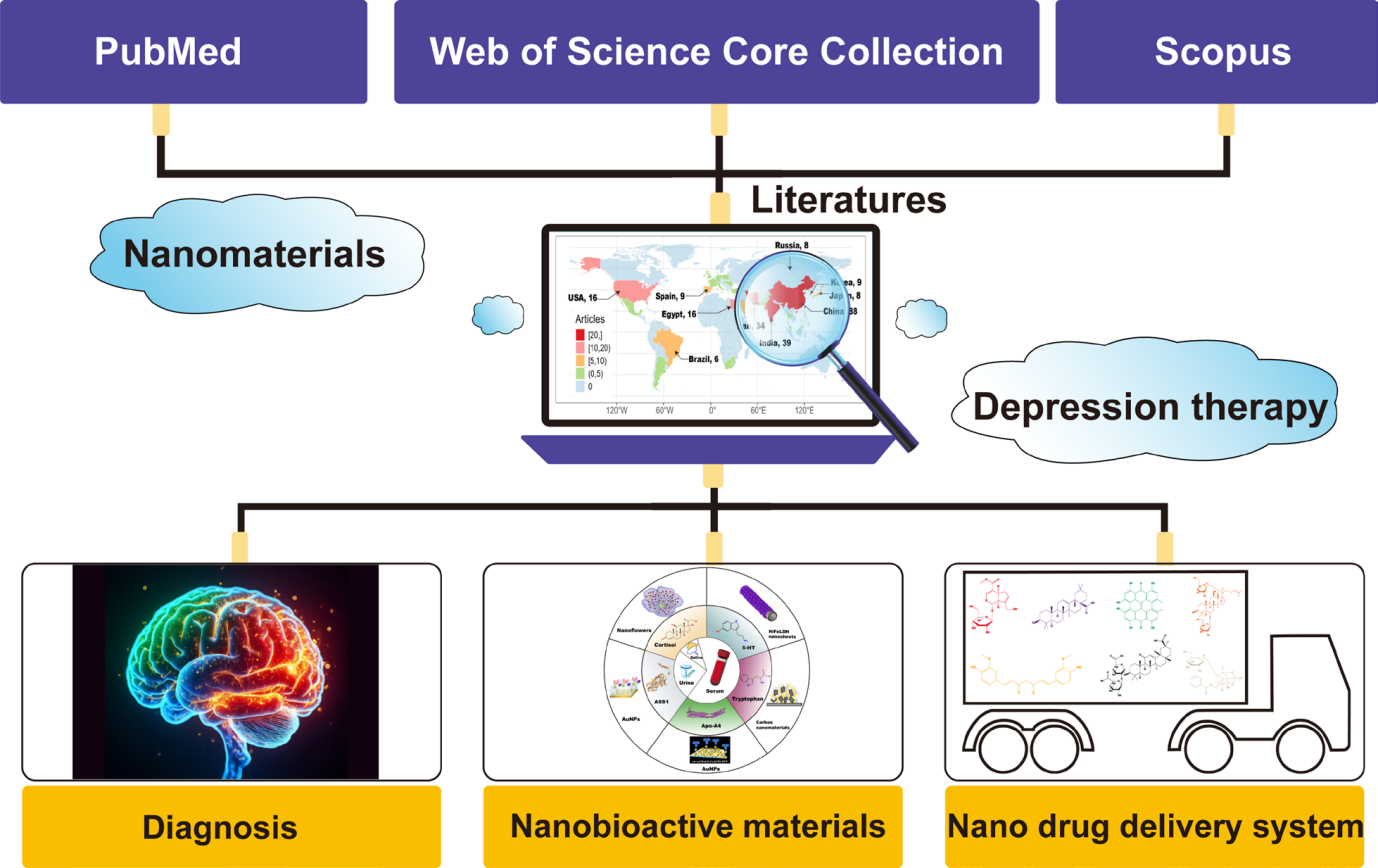

## Introduction

Depression is a common and severe mental disorder characterized by significant and persistent low mood, loss of interest, fatigue, cognitive decline, and suicidal tendencies. Its pathogenesis is complex and involves multiple factors including genetic susceptibility, neurotransmitter system dysregulation, neuroendocrine abnormalities, and neuroinflammatory responses [1, 2]. According to a report by the World Health Organization, depression is a major cause of disability worldwide. It affects approximately 322 million individuals and accounts for 7.5% of all years lived with disability worldwide [3]. Current treatments include medications, such as selective serotonin reuptake inhibitors (SSRIs), and psychological interventions; however, several challenges, such as delayed onset of action, ineffectiveness in some patients, and significant systemic side effects, highlight the urgent need for new antidepressant strategies.

Nanotechnology has revolutionized the diagnosis and treatment of depression in two ways. First, certain nanomaterials, such as cerium oxide nanoparticles and polydopamine nanoparticles, have been found to possess inherent antidepressant potential. They ameliorate depressive-like behaviors through antioxidant, anti-inflammatory, and neuroprotective effects [4–6]. Second, the efficient delivery of antidepressants using a nano drug delivery system (NDDS) can significantly improve drug solubility, stability, and bioavailability; facilitate drug delivery across the blood‒brain barrier (BBB); and enable targeted and controlled release, thereby enhancing antidepressant effects while mitigating side effects [7–9]. These advances demonstrate that nanomaterials not only enhance drug delivery efficiency but also provide new insights into the treatment of depression through the synergistic regulation of multiple mechanisms.

To comprehensively understand the dynamics and development trends of nanomaterials in the field of depression, this article systematically reviews relevant literature using scientometrics. By quantitatively analyzing metrics, such as annual publication volume, country, institution, journal, and keywords, scientometrics objectively reveals research hotspots, development trajectories, and key contributors within the field. This effectively overcomes the subjective limitations of traditional reviews and provides an objective scientometrics-driven perspective. For example, researchers have used scientometrics to conduct research in the field of intranasal drug delivery and explored several challenges faced by researchers in this area, including drug entry and elimination mechanisms, drugs compatible with the nasal cavity, dosage forms to overcome limited drug loading and poor solubility, and diseases for which intranasal delivery is a suitable approach [10]. Pharmaceutical researchers have used scientometrics to study the small molecule genipin and demonstrate its diverse therapeutic effects, including anti-inflammatory, antioxidant, anticancer, hepatoprotective, antidiabetic, antidepressant, and neuroprotective properties [11]. With respect to pharmaceutical formulations, researchers have used scientometrics to investigate the biopharmaceutical applications of genipin cross-linked hydrogels. Based on their scientometric findings, they explored the future research and development of genipin cross-linked hydrogels in food science, drug delivery, tissue engineering, and wound healing [12]. Despite the rapid development of research on the application of nanomaterials in the treatment of depression, scientometric reviews are still lacking.

In this study, scientometrics was used to analyze the current status of nanomaterials in depression therapy. By analyzing annual publication volume, country, institution, journal, and keywords, current progress in the use of nanomaterials for depression therapy was identified. Keyword analysis was performed to assess and discuss current focus areas of research, including diagnosis, treatment, nanomaterials with antidepressant effects, and NDDS in nanomaterials for depression therapy. In summary, this article presents a scientometric analysis that provides new insights and perspectives on the use of nanomaterials for depression therapy and indicates a pathway for subsequent research and development.

## Scientometric analysis

### Data retrieval, download, analysis and visualization

Data retrieval was performed until December 31, 2025. The data sources and download formats are listed in Table 1. The sources include databases such as Web of Science Core Collection (WOSCC), PubMed, and Scopus. A document listing the article types and whether they are in English was created and used for further analysis. Finally, literature data in the corresponding format were downloaded.

**Fig. 1:**
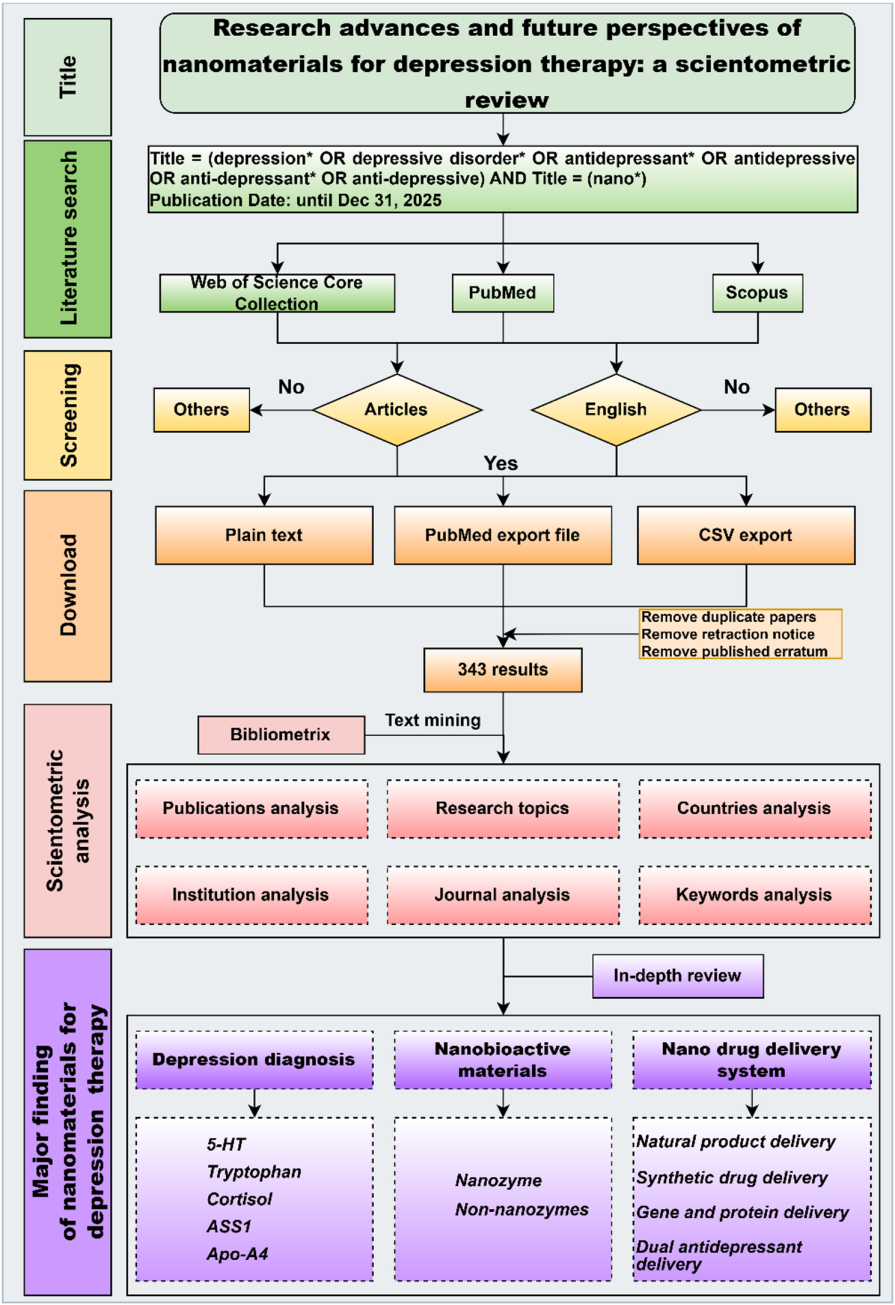
Scheme of the scientometric analysis procedure

Literature data were analyzed and visualized primarily using Bibliometrix (version 4.0.0), tidyverse (version 1.3.2), and VOSviewer (version 1.6.18), which included publications, categories, countries, institutes, journals, and keyword analysis. A schematic of the scientometric analysis procedure is shown in Fig. 1.

**Table 1:** Source and download format of literature data

Rank	Source	Advanced search	Document type	Results	Format (extension)
1	Web of Science Core Collection (https://www.webofknowledge.com/)	TI = (depression* OR depressive disorder* OR antidepressant* OR antidepressive OR anti-depressant* OR anti-depressive) AND TI = (nano*)	Article, English	296	Plaintext (.txt)
2	PubMed (https://pubmed.ncbi.nlm.nih.gov/)	depression*[Title] OR depressive disorder*[Title] OR antidepressant*[Title] OR antidepressive[Title] OR anti-depressant*[Title] OR anti-depressive[Title]) AND (nano*[Title]	English	188	PubMed export file (.txt)
3	Scopus (https://www.scopus.com/)	(TITLE ( depression*) OR TITLE (depressive disorder*) OR TITLE (antidepressant*) OR TITLE (antidepressive) OR TITLE (anti-depressant*) OR TITLE (anti-depressive)) AND TITLE (nano*)	Article, English	312	CSV export (.txt)

### Characteristics and annual growth of publications

The number of papers published each year showed that research on the use of nanomaterials for depression therapy can be classified as the initial exploration period (1977–2014), stable growth period (2015–2020), and explosive growth period (2021–2025) (Fig. 1A) [13]. During the 37 years between 1977 and 2014, the number of papers published annually in this field remained at single-digit levels. This stage shows obvious characteristics of sporadic exploration with large fluctuations between years, reflecting that the cross-integration of nanomaterials and neuropsychiatric diseases, especially research on depression, was still in a very early stage of concept verification and technical exploration. This stage of research focuses mainly on the discussion of basic principles, preliminary in vitro experiments, or simple animal model verification, and represents a phase where a large-scale research hotspot has not yet been formed. The year 2015 was an important turning point. The annual number of papers exceeded double digits for the first time, reaching 17 papers, which was a significant increase from the previous year. From then until 2020, the annual number of papers remained stable, between 17 and 28. The significant growth during this period indicated that the field had begun to receive widespread attention and recognition from the research community. Possible reasons for this include the developments in nanomaterial synthesis and characterization technology, a detailed understanding of the BBB penetration mechanism, and the initial confirmation of the great potential of nanocarriers for drug delivery. The slight decline in 2018 may reflect fluctuations in research cycles or specific years. However, the overall upward trend remained unchanged. Since 2021, research in this field has grown rapidly. The number of publications in 2021 reached 30, nearly 1.57 times greater than in 2019. After a slight adjustment in 2022, the number of publications in 2023 surged to 47, reaching a historical peak. This rapid growth over the past 5 years, especially the surge in 2023, strongly indicates that nanomaterials have become a very dynamic and promising frontier in research related to the treatment of depression.

### Distribution of research topics

The distribution of research topics based on WOSCC revealed distinct interdisciplinary characteristics and a current research focus on nanomaterials for depression therapy. This cross-disciplinary research field is dominated by three pillars—materials science, nanotechnology, and pharmacy/chemistry (Fig. 1B). Specifically, “Materials science, multidisciplinary” (52 articles), “Nanoscience & nanotechnology” (48 articles), and “Pharmacology & pharmacy” (48 articles) constituted the largest share of publication volume, significantly greater than that of other fields. This clearly revealed that the development of new nanomaterials, methodological innovations in nanotechnology, and the design and evaluation of NDDS for depression therapy were the core driving forces and research focuses. The fields that followed are “Chemistry, multidisciplinary” (39 articles), “Physics, applied” (37 articles), and “Chemistry, analytical” (36 articles), highlighting the basic supporting role of chemistry in the synthesis and characterization of nanomaterials and that of physics in the research regarding nanodevices or delivery mechanisms. Another branch of physics, “Physics, condensed matter” (19 articles), which involves the application of special physical properties of nanomaterials (such as magnetism for targeting or imaging) for neuromodulation or diagnosis, also has a certain share of publications. Notably, the contributions of core life sciences and medical disciplines are relatively weak in terms of publications directly related to depression. The number of publications in “Neurosciences” (8 articles) and “Medicine, research & experimental” (5 articles) was much lower than that in the fields of materials and pharmacy. This research area focused on the construction of technology platforms (nanocarriers and delivery systems) and in vitro/preliminary in vivo verification stages, whereas detailed explorations of the specific effects of nano-interventions on complex neural circuits and molecular mechanisms of depression (neurotransmitters, neurotrophic factors, inflammation, etc.), as well as rigorous preclinical model verification and translational medicine research, are still insufficient or in the early stages. Similarly, contributions of “Biochemistry & molecular biology” (9 articles) and “Biochemical research methods” (5 articles) are relatively limited, suggesting that there is still significant scope for systematic elucidation of the interaction mechanism between nanomaterials and nerve cells/brain tissues at the molecular level.

### Most productive countries, institutions, and journals

**Fig. 2:**
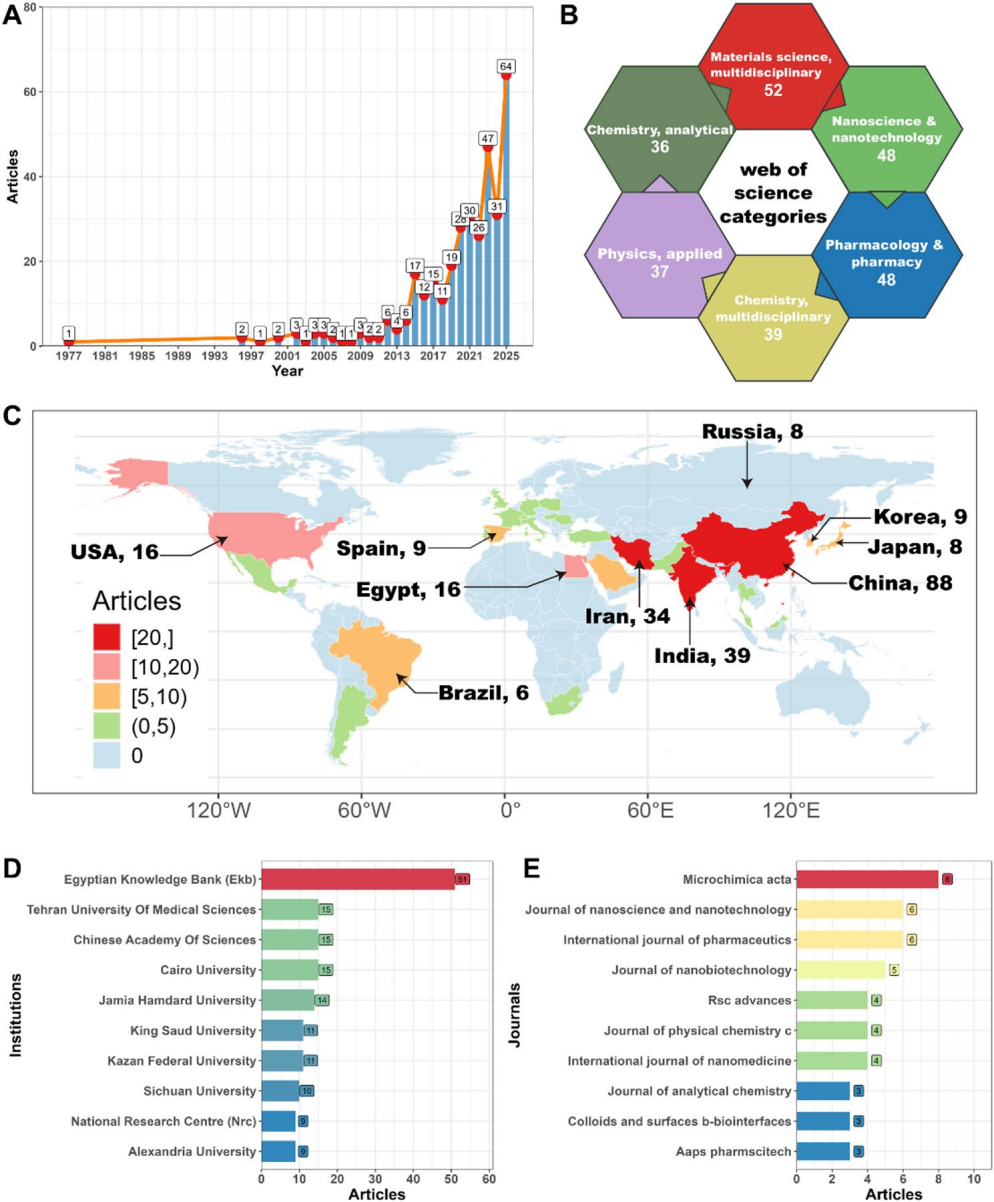
Comprehensive analysis of scientometrics. (**A**) Publications characteristics and annual growth. (**B**) Distribution of research topics. Most productive countries (**C**), institutions (**D**), and journals (**E**)

In terms of distribution across countries, China holds a significant lead with 88 papers, which exceeds the total number of papers published by second-ranked India (39 papers) and third-ranked Iran (34 papers), and accounting for 37.8% of the total number of papers published by the top 10 countries (233 papers) (Fig. 2C). This significant lead may be because of China’s strategic investments in nanotechnology for depression therapy, such as the national key project of NanoResearch, and the large size of its scientific research team. Notably, the United States, a traditional scientific research powerhouse, ranked fourth with 16 papers, tied with Egypt. This is far lower than the performance of the United States in conventional biomedical research, suggesting that their research and development focus may be on other directions of depression intervention (such as neuromodulation or gene therapy) or more on the clinical translation of nanomaterials rather than basic research. Asian countries are showing a collective rise: India, Iran, South Korea (nine papers), and Japan (eight papers) account for 38.6%, reflecting enthusiasm for research and development in this field in Asia. In particular, the high number of publications from India and Iran is related to their recent funding policies aimed at strengthening nanomaterials for neurological diseases. Egypt, the only African country on the list (16 papers), may benefit from cross-border cooperation projects. European countries performed relatively well, with Spain (nine papers) in the top ten. The inclusion of Russia (eight papers) and Brazil (six papers) shows that emerging economies are actively developing this cross-cutting field. The low rankings of Japan and South Korea (eighth and ninth) are worth noting, perhaps because their research on depression focuses more on traditional pharmacology and social psychology.

Figure 2D shows the main contributing institutions worldwide and patterns of their scientific research output on nanomaterials for depression therapy. In terms of the number of papers published by institutions, the Egyptian Knowledge Bank ranked first, with 51 papers related to nanomaterials for depression therapy. Its holds a significant lead over other institutions, with more than three times the number of papers published by the second-ranked institution. A total of three institutions were tied in second place—Cairo University, Chinese Academy of Sciences, and Tehran University of Medical Sciences—with 15 papers each. Jamia Hamdard University ranked fifth with 14 papers. Kazan Federal University and King Saud University published 11 papers each and tied for sixth place. Sichuan University published 10 papers, ranking eighth. Alexandria University and the National Research Centre each published nine papers each, tied for ninth place. The Egyptian Knowledge Bank is far ahead with 51 papers, and Cairo University (15 papers), Alexandria University (nine papers), and the National Research Centre (nine papers) are also among the top ten. The high productivity of the Egyptian Knowledge Bank, Egypt’s national knowledge platform, may be because of the integration of the results of many universities and research institutions across Egypt, reflecting the country’s key investment or strategic layout in nanomaterials for depression therapy. The geographical distribution of the top 10 institutions shows a globalization and diversification trend. In addition to Egypt, other institutions are from China, Iran, India, Russia, and Saudi Arabia. These results show that in the emerging cross-cutting research field of nanomaterials for depression, countries and regions, such as Egypt, China, Iran, India, Russia, and Saudi Arabia, that are emerging research hubs, are playing a leading role, actively investing in and achieving significant results.

An analysis of the top 10 English journals and the number of articles on nanomaterials for depression research (Fig. 2E) revealed that the research results presented a relatively scattered but essentially core publication pattern. *Microchimica Acta* ranked first with eight articles, followed by *International Journal of Pharmaceutics* and *Journal of Nanoscience and Nanotechnology*, with six related studies each. *Journal of Nanobiotechnology* ranked fourth with five articles. *International Journal of Nanomedicine*, *Journal of Physical Chemistry C*, and *RSC Advances* published 4 studies each, jointly ranking fifth. *AAPS PharmSciTech*, *Colloids and Surfaces B*: *Biointerfaces*, and *Journal of Analytical Chemistry* published three articles each and were ranked a joint eighth. The knowledge output on nanomaterials for depression research is primarily concentrated in professional journals related to materials science, pharmacy, analytical chemistry, and nanobiotechnology. Research hotspots are concentrated on the development of NDDS and establishment of related bioanalytical technologies. The overall number of articles in each journal is not high, and the distribution is relatively scattered, indicating that this interdisciplinary field is still in a stage of rapid development; however, it has not yet matured and formed a significant scale in the top journals. Importantly, this journal list provides a valuable reference for researchers to track progress in the field and select submission targets.

**Fig. 3:**
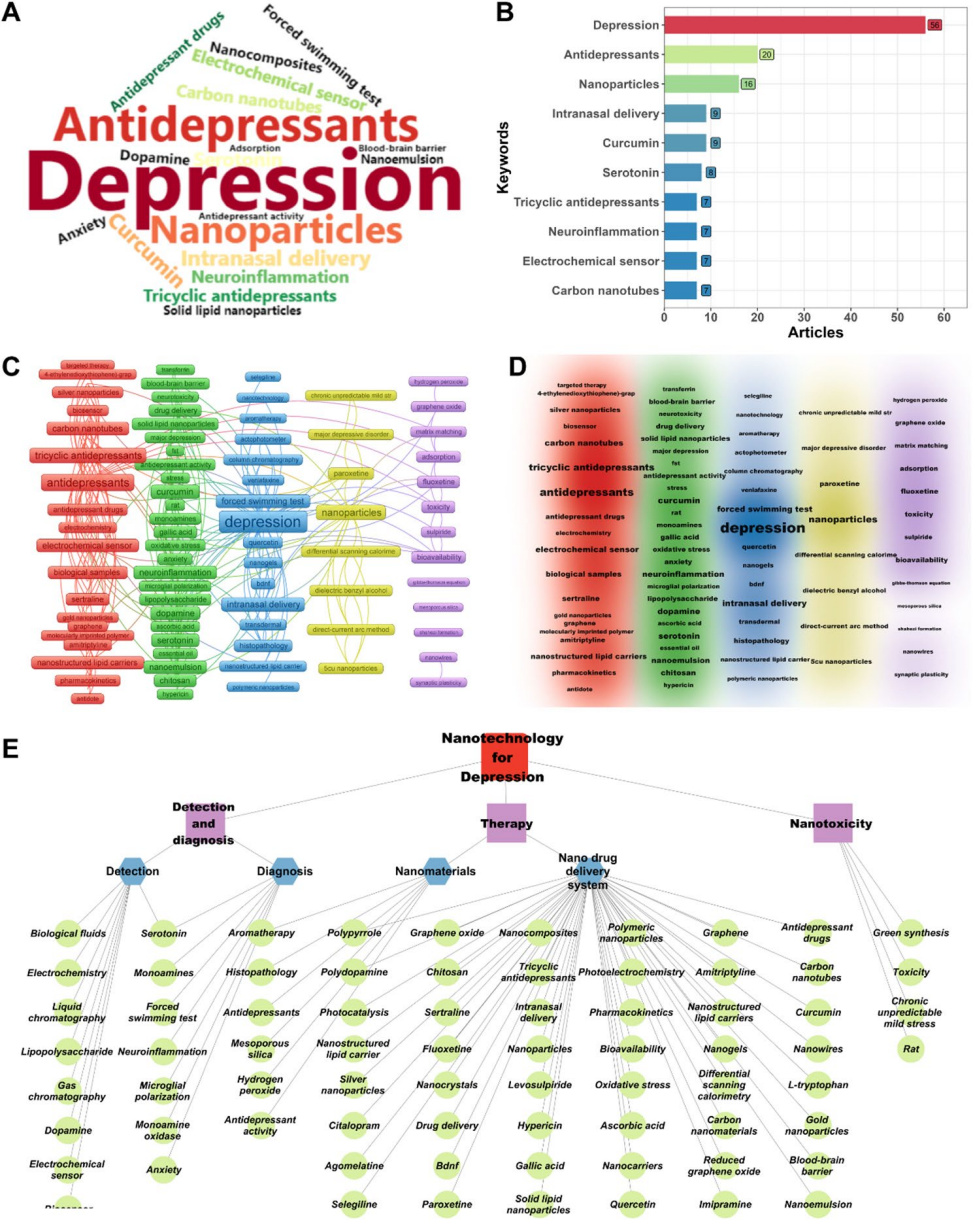
Keyword analysis. (**A**) Word cloud of the top 20 keywords. (**B**) Bar chart of the top 10 keywords. (**C**) Keyword cluster analysis based on VOSviewer. (**D**) Density visualization of keywords based on VOSviewer. (**E**) Keyword cluster analysis based on manual analysis

### High-frequency keyword analysis

Important keywords are shown in the word cloud of the top 20 keywords (Fig. 3A) and bar chart of the top 10 keywords (Fig. 3B). Unsurprisingly, “Depression” is the absolute core keyword, establishing the foundation of the entire field. It is closely followed by “Antidepressants”, which clearly reveals that the primary driving force and ultimate goal of research in this field is to use nanomaterials to improve the efficacy of existing antidepressants or develop new nano antidepressant therapies. This core goal is common to most of the high-frequency keywords. Nanomaterials and technologies themselves, including “Nanoparticles”, “Carbon nanotubes”, “Nanocomposites”, “Nanoemulsion”, and “Solid lipid nanoparticles” are key means to achieve this goal. The core advantage of these carrier technologies lies in overcoming the bottleneck of traditional drug delivery, especially by breaking through the BBB, which is a key challenge in the treatment of central nervous system (CNS) diseases. Among them, “Intranasal delivery”, a noninvasive, targeted drug delivery route that can effectively bypass the BBB, has received significant attention. In addition, research also delved into specific drug categories, such as “Tricyclic antidepressants”. Further, the “Forced swimming test”, which is the key animal behavioral model for evaluating drug efficacy, and “Antidepressant activity”, which is the core pharmacological indicator, were other important keywords. In terms of neurobiological mechanisms, the classic monoamine neurotransmitters “Serotonin” and “Dopamine” are still the main research objects, reflecting the continued attention given to traditional pathological mechanisms of depression. Moreover, the emergence of “Neuroinflammation” shows that the research on new pathological mechanisms of depression on the basis of the theory of neuroinflammation has been incorporated into the development of nano-therapy strategies. In addition, the high frequency of “Curcumin”, a natural compound, highlights the special research direction of using nanomaterials to improve its bioavailability, ability to cross the BBB, and potential antidepressant and anti-inflammatory effects. The emergence of “Electrochemical sensor” and “Adsorption” indicates that the high sensitivity and specificity of nanomaterials (such as carbon nanotubes) can be used to develop advanced diagnostic tools for detecting depression-related biomarkers such as neurotransmitters. The correlation of the keyword “Anxiety” reflects the high comorbidity of depression and anxiety disorders, and nanomaterials research also focuses on joint interventions for these two closely related symptoms.

### Keywords cluster analysis

Through clustering of the bibliometrix and visualization using VOSviewer, five core research directions were identified (Fig. 3C and D): nanosensing and drug detection technology (Cluster 1), nanocarriers and neuromodulation (Cluster 2), new drug delivery routes and drug efficacy optimization (Cluster 3), specific drug‒material combination research (Cluster 4), and nanomaterial biological effects and safety (Cluster 5). Nanosensing and drug detection technology focuses on nanomaterial-driven antidepressant detection technology; with carbon nanotubes, graphene/reduced graphene oxide, and silver/gold nanoparticles as core materials; molecularly imprinted polymers; electrochemical sensors/biosensors; gas/liquid chromatography; and high-sensitivity detection of antidepressants in biological samples/fluids. Keywords such as “solid-phase extraction,” “microextraction,” and “pharmacokinetics” further highlight the importance of monitoring drug metabolism. Nanocarriers and neuromodulation focus on the neuroprotective mechanisms of NDDS, which involves lipid carriers, natural ingredients, and polymers. The keyword cluster revealed some major research focus areas—targeted delivery, pathological intervention, animal model verification, new drug delivery routes, and drug efficacy optimization—highlighting the nanomodification of non-oral drug delivery systems. Technical paths include nanogels and lipid carriers, and their efficacy has been verified using animal behavior and neurochemical indicators. Research on specific drug‒material combinations presents highly specialized research topics, such as the combination of “5Cu nanoparticles” and “paroxetine” for “major depressive disorder,” combined with “chronic unpredictable mild stress” and material characterization technology. This review highlights the exploration of the synergistic mechanisms between nanomaterials and specific antidepressants. The biological effects and safety of nanomaterials depend on their biocompatibility. Mesoporous silica and graphene oxide are used to improve drug bioavailability; however, attention has been paid to their toxicity and synaptic plasticity. The keywords “adsorption” and “hydrogen peroxide” suggest correlations between the surface and interface properties of materials and their biological effects.

Furthermore, manual analysis using software (Fig. 3E) revealed that nanomaterials are used for various aspects, such as detection, diagnosis, therapy, and nanotoxicity, in depression. Detection and diagnostic methods mainly consist of biosensors such as electrochemical sensors, gas chromatography, electrochemistry, and liquid chromatography; key substances such as serotonin, dopamine, and monoamine oxidase; the gold standard for detecting depression, such as forced swimming tests; and pathological characteristics such as neuroinflammation and microglial polarization. Nanotechnology therapy for depression primarily involves the use of nanomaterials and NDDS for the treatment of depression. NDDS include natural drug delivery, synthetic drug delivery, gene/protein delivery, and dual antidepressant drug delivery. The important findings regarding the use of nanomaterials for depression therapy, obtained by combining software and manual analyses, are presented and further discussed below.

### Major findings of scientometric analysis

#### Nanomaterials for the diagnosis of depression

**Fig. 4:**
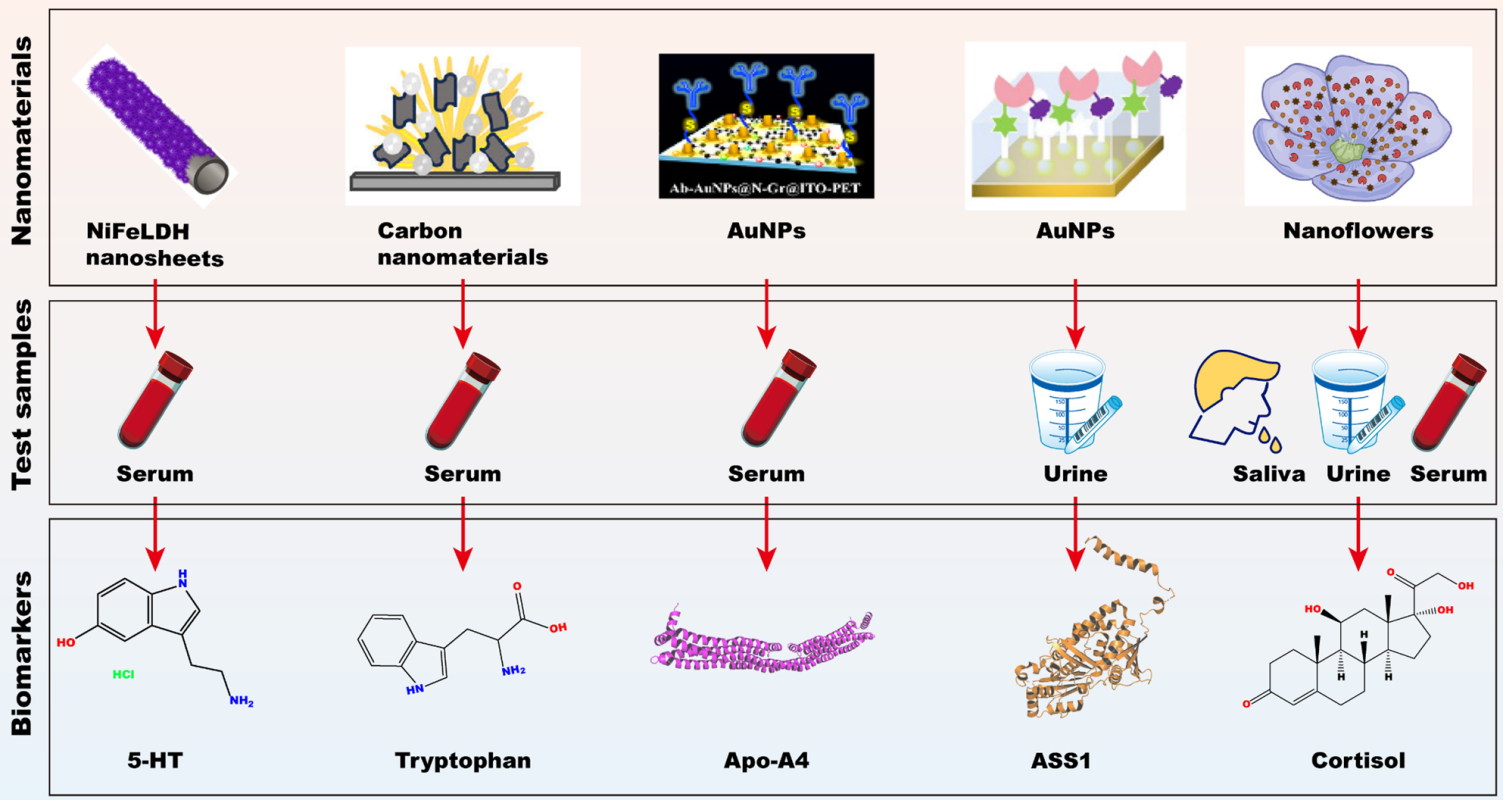
Nanomaterials for detection of biomarkers in depression therapy

The clinical diagnosis of depression primarily depends on depression scales and subjective assessments by psychiatrists, resulting in substantial rates of missed and misdiagnosis owing to the absence of objective diagnostic indicators. Disease-marker objectivity enhances the significance of monitoring index values in supporting diagnosis and delivering predictive disease-related information. Therefore, early warning, objective diagnosis, and prognostic treatment of depression are critical for screening and sampling non-invasive and readily accessible depression markers using robust and practical analytical methods. Currently, key biomarkers include 5-hydroxytryptamine (5-HT; also known as serotonin), tryptophan, cortisol, argininosuccinate synthetase 1 (ASS1), and apolipoprotein A4 (Apo-A4). However, these biomarkers are difficult to detect because of the low concentrations and complexities of their components in the body. The application of nanomaterials, which may enhance the detection of low concentrations of depression-associated biomarkers in the body, provides a promising strategy for the diagnosis of depression (Fig. 4).

*5-HT*.

5-HT is a neurotransmitter and hormone that regulates physical, biochemical, and psychological functions, including sleep, sexual activity, anxiety, and aggressiveness. Previous studies have suggested that healthy individuals have typical blood serotonin concentrations ranging from 500 nM to 1.7 µM. Low 5-HT levels can result in depression and anxiety. Therefore, early diagnosis of 5-HT abnormalities is crucial for diagnostic and biological research on depression. Owing to their complexities, the present analytical methods are restricted in their capacity to provide ultralow detection. As a result, researchers have investigated lower-level sensitive electrochemical detection of 5-HT by using the synergy between carbon nanotubes (CNTs) and layered double hydroxide (LDH). The modified NiFeLDH/N-doped CNTs exhibit better electrochemical activity and high sensitivity and span a concentration range of 0.01–400 µM, with a detection limit of 1.2 nM owing to the significantly high electrochemically active surface area, remarkable conductivity, and good stability. The recovery rates achieved in the human urine and serum samples were 96.0–96.8% and 96.7–97.2%, respectively, which are very impressive as they are close to the high-performance liquid chromatography (HPLC) data, indicating the feasibility of this method for real-time monitoring. The operational stability of the proposed sensor reached 89.13% after 6 weeks [14]. In addition, another study used disposable screen-printed electrodes for the detection of depression biomarkers, which exhibited a linear response in the dynamic concentration range of 0.1–30 µM, with a detection limit of approximately 60 nM [15]. The sensor response was reproducible from electrode to electrode, and no deactivation or surface fouling of the sensor was observed across several experimental measurements. The present study highlights that nanotechnology-based materials display good anti-interference ability and excellent storage stability, achieving low-cost detection of 5-HT in blood serum, urine, and saliva samples.

## Tryptophan

Tryptophan (Trp) is one of the eight major amino acids. Although it is required only in limited quantities, stable levels of Trp are necessary for the control of neuronal and immune activities and play an indispensable role in the functioning of the human body. Trp plays a key role in human mental health disorders and is strongly associated with the development of depression, and its levels are negatively correlated with depression scores. Tests for Trp levels can assist doctors in screening, diagnosing, and monitoring the mental status of patients. Hence, the detection of Trp in the human body is highly important for healthcare systems. Researchers prepared Co_3_O_4_ nanoflowers via hydrothermal methods using nickel foam (NF) with a porous structure as the substrate, and Co_3_O_4_/reduced graphene oxide (rGO)/TiO_2_/NF composites were created by combining rGO and TiO_2_ [16]. The Co_3_O_4_ nanoflowers provide a large specific surface area, rGO improves the electrical conductivity and stability of the composites, and the photocatalyst TiO_2_ further improves the electrical conductivity and density of the electrochemically active sites of Co_3_O_4_/rGO/NF. The nanostructure composites exhibit high Trp-sensing activity at low concentrations, with detection limits calculated in the linear ranges of 0.01–30 µM and 30–80 µM of 0.004 versus 0.006 µM, respectively, and sensitivities of 1469.86 and 929.47 µA·µM^− 1^·cm^− 2^, respectively. The combined effect of these three factors led to excellent performance of the nanomaterial in the detection of low concentrations of Trp. The Co_3_O_4_/rGO/TiO_2_/NF nanostructure composites displayed excellent performance in the detection of low concentrations of Trp and were beneficial for screening and diagnosing the occurrence and development of depression. Moreover, nanomaterials present excellent characteristics for the simultaneous determination of the depression biomarkers 5-HT and Trp [17]. The SnO_2_–SnS_2_-modified glassy carbon electrode nanomaterials exhibited high electrocatalytic activity, sensitivity, and selectivity toward the oxidation of 5-HT and Trp, with a wide linear range (0.1–700 µM for 5-HT and 0.1–800 µM for Trp), low detection limits (45 and 59 nM), good reproducibility, repeatability, stability, and anti-interferent ability at physiological pH. Hence, the developed SnO_2_–SnS_2_ nanocomposite could provide a potential platform for the analysis of 5-HT and Trp in clinical and diagnostic research.

### Cortisol

Cortisol is not only a steroid hormone that is the end-product of the central stress response system but is also a key indicator for the diagnosis and treatment of depression. Precise analysis of cortisol helps assess stress and depression objectively, avoiding subjective factors using self-assessment scales to evaluate stress and depressive status. Owing to the strong correlation among cortisol, stress, and depression, a convenient and noninvasive method to monitor cortisol can be very useful for stress assessment. All-in-one calcium nanoflowers (CaHPO_4_-AM-HRP-SA NFs) have been synthesized to develop a simple but powerful biosensor for cortisol detection. Compared with free enzymes, the high specific surface area and allosteric modulator provided by hybrid nanoflowers, which have inherent advantages, significantly increase catalytic ability and stability [18]. The CaHPO_4_-AM-HRP-SA NFs also endow the sensor with two output signals from one sample, allowing the as-prepared sensor to undergo self-calibration. The sensor exhibited simplicity and excellent sensitivity with a low limit of detection (LOD) of 98.5 pg·mL^− 1^. A sensitivity evaluation of cortisol in rat serum, human urine, and saliva samples was performed, and the antidepressant effects of different medications were assessed. Therefore, nanotechnology-based materials can measure trace amounts of cortisol in human urine and saliva, which vary greatly between individuals.

### ASS1

Compared with that in healthy subjects, the content of human arginine succinate synthase 1 (ASS1), an arginine cycle enzyme, in the urine of patients with depression is lower; therefore, ASS1 can be used as a marker of depression. Currently, quantitative detection of ASS1 is mainly based on enzyme-linked immunosorbent assay (ELISA) and western blotting (WB). These two methods have high reliability; however, their high detection cost, slow detection speed, use of toxic reagents, and other drawbacks make it difficult for these techniques to be widely adopted in the detection of clinical markers in large sample volumes. Therefore, researchers have developed highly sensitive, highly selective, and low-cost quantitative gold nanoparticle–based dual-epitope peptide–imprinted sensors for the urinary depression marker ASS1 [19]. The sensor had a relatively high sensitivity and presented a linear range of 0.15–6000 pg·mL^− 1^ with an LOD of 0.106 pg·mL^− 1^. The sensor exhibited good reproducibility, repeatability, stability, and good selectivity. Moreover, the sensor also exhibited good recovery in urine samples. This is the first highly sensitive and selective electrochemical assay for the depression marker ASS1 in urine, and is expected to enable the noninvasive and objective diagnosis of depression.

### Apo-A4

Apolipoprotein A4 (Apo-A4), a biomarker, plays a crucial role in regulating various physiological and emotional/behavioral responses to stress exposure. Apo-A4 is closely related to the occurrence and development of depression and has been studied by many researchers in recent years. Therefore, achieving an accurate, efficient, and convenient testing method for Apo-A4 is extremely important for early screening and confirmation of depression and other related psychiatric disorders, as well as for monitoring and controlling the disease course. N-Gr and AuNPs have been used as nanomaterials to prepare AuNPs@N-Gr@ITO-PET electrodes that can perform as flexible electrochemical immunosensors owing to their high selectivity, sensitivity, and stability in the detection of the depression marker Apo-A4 [20]. The fabricated immunosensor showed good linearity over a wide range of 0.023–300.00 ng/mL with a low detection limit of 0.010 ng/mL when used for the determination of Apo-A4 in 100% whole serum samples. The flexibility of the constructed immunosensor and the stability and sensitivity of biomolecular analysis are expected to be further improved to enable the fabrication of implantable depression marker in situ monitoring probes with the help of micromachining technology, which will lead to the development of a promising method for the objective, efficient, and accurate clinical diagnosis of depression.

### Nano-bioactive materials for depression therapy

#### Nanozyme materials

Nanozymes, artificial enzyme mimics that combine the properties of nanomaterials with enzyme-like catalytic functions, are composed of metals, metal oxides, or carbon-based nanomaterials [21]. A critical factor in the pathology of depression is inactivation of tryptophan hydroxylase, which reduces 5-HT synthesis. Fang et al. demonstrated that Fe₃O₄ nanoparticles mimic tryptophan hydroxylase activity, catalyzing the conversion of tryptophan into 5-HT within stressed neurons, thereby compensating for impaired tryptophan hydroxylase function in the brain. This process relies on elevated endogenous levels of ascorbic acid and hydrogen peroxide [22]. Recent studies have also shown that nanozymes, including those that scavenge reactive oxygen species (ROS) and reactive nitrogen species (RNS) (·OH, O_2_^−•^, H_2_O_2_, ONOO^−^), and oxygen/nitrogen-centered free radicals, have outstanding antioxidant activities for depression therapy. Hu et al. developed a nanozyme consisting of cerium oxide–modified Pd nanosheets encapsulated in red blood cell (RBC) membrane (RBC-Pd-CeO_2_) nanosheets [4]. The RBC-Pd-CeO_2_ nanosheets exhibited significant ROS and RNS clearance through the activities of antioxidant enzymes, such as superoxide dismutase and catalase, and further reduced the proliferation of microglia and astrocytes, thereby alleviating neuroinflammation and depression-like behaviors. Jia et al. reported an N-doped carbon dot nanozyme (CDzyme) that provided abundant electrons, hydrogen atoms, and protons for reduction reactions, as well as catalytic sites to mimic redox enzymes [23]. CDzymes markedly improved depression-like behaviors, restored hippocampal neurotransmitter levels, and normalized the gut microbiota composition and amino acid metabolism, highlighting their therapeutic potential via the gut–brain axis.

### Non-nanozymes materials

In addition to nanozymes, non-nanozymes materials containing magnesium and selenium are promising novel treatment strategies for depressive disorders. Li et al. designed novel low-toxicity, anti-inflammatory magnesium hydride (MgH_2_) nanoparticles [24]. MgH_2_ nanoparticles alleviate depression-like behaviors in acute restraint stress models by promoting microglial M2 polarization, inhibiting M1 polarization, and reducing oxidative stress and mitochondrial damage. Wang et al. prepared Se nanoparticles to supplement Se in the brain, thereby improving depression [25]. Se nanoparticles modulate dopamine (DA) content, alter microglial morphology (e.g., ramification index and solidity), and trigger neuroinflammatory responses via p-STAT3 nuclear translocation. Ultimately, they repair neural function and reduce depression-like symptoms.

In general, nano-bioactive materials can exert antidepressant effects through neurotransmitter regulation, antioxidant barrier construction, and gut–brain axis regulation, indicating their strong antidepressant potential.

### Nano drug delivery system for antidepressant delivery

#### Natural product delivery

Natural products are organic or inorganic compounds derived from plants, animals, or microorganisms through primary or secondary metabolic processes with minimal artificial synthesis or chemical alteration [26]. These substances are valued in drug development because of their natural origin, generally favorable safety profiles, structural diversity, synergistic interactions between components, environmental compatibility, and potential for sustainable production [27]. However, the therapeutic applications of certain natural antidepressants are limited by their suboptimal physicochemical characteristics, including poor water solubility, high molecular weight, and susceptibility to hydrolysis. These limitations often result in reduced bioavailability and diminished efficacy. NDDS have emerged as promising carriers to overcome these challenges. Nanomaterials can significantly augment the antidepressant effects of natural compounds by improving drug stability, enhancing bioavailability, enabling targeted delivery to specific proteins or neural pathways, and facilitating BBB penetration [28]. As summarized in Table 2, various nanocarriers—such as nanovesicles, cubic liquid crystalline nanoparticles, nanosheets, nanorods, nanoemulsions, graphene oxide–based nanocarriers, and polysaccharide nanogels—have been successfully employed to deliver natural products with antidepressant activities [29–35]. Notable examples include geniposide, oleanolic acid, hypericin, lemon and bergamot essential oils, curcumin, ginsenoside Rg1, glycyrrhizic acid, and paeoniflorin, all of which exhibit improved efficacy when formulated using these advanced systems. Natural products have emerged as important resources for drug development because of their natural origin and multiple advantages [36]. However, some natural antidepressant products have issues such as poor solubility and stability. NDDS significantly enhance the antidepressant effects by improving the stability, bioavailability, and brain-targeting abilities, enhancing the possibility for clinical translation.

**Table 2:** Nano drug delivery system for natural product delivery

Nano type	Main components	Natural products	Function	Antidepressant effects/mechanisms
Nanovesicles [29]	Polyvinylpyrrolidone, CuCl_2_, NaBH_4_, selenium	Geniposide	Targeted and inhibited P2ry12	λ Downregulated P2ry12 expression λ Improved neuronal activity λ Reduced cell apoptosis λ Decreased inflammation and ROS levels
Cubic liquid crystal nanoparticle [30]	Phytantriol	Oleanolic acid	Enhanced brain-targeting efficiency	λ Affected IL-4, IL-6, acetylcholinesterase, acetylcholine, 5-HT, and brain-derived neurotrophic factor (BDNF) levels λ Improved sucrose preference, forced swimming, and box shuttle tests
Nanosheets [31]	RVG29-PEG-NH_2_, black phosphorus	Hypericin	Traversed BBB	λ Alleviated depressive symptoms λ Reduced oxidative stress levels
Nanorods [32]	Mesoporous silica	Lemon and bergamot essential oil	Sustained released of essential oils	λ Increase monoamine neurotransmitters in the brain
Nanoemulsion [37]	Demethoxycurcumin, bisdemethoxycurcumin	Curcumin	Enhance drug stability and bioavailability	λ Improved the depressant-like behaviors λ Elevated 5-HT content in plasma and brain
Graphene oxide nanocarrier [34]	Borneol, graphite powder, PEG	Ginsenoside Rg1	Opened tight junctions and inhibited the efflux system of BBB	λ Improved the depressant-like behaviors
Polysaccharide nanogel [35]	Transferrin, sodium alginate, disulfide bonds	Glycyrrhizic acid, paeoniflorin	Crossed the BBB and response to ROS in brain	λ Improved the depressant-like behaviors λ Regulated ROS-induced MAPK and NLRP3 signaling pathways

### Synthetic drug delivery

Synthetic drugs are pharmacologically active substances artificially prepared in a laboratory or factory using chemical or semi-synthetic methods. Synthetic drugs have advantages such as a well-defined composition, high purity, controlled quality, and greater amenability to large-scale production. However, their disadvantages include complex molecular structures, numerous synthesis steps, and high production costs. More importantly, although many synthetic drugs exert therapeutic effects, they are also accompanied by a series of side effects, such as adverse reactions in the nervous, cardiovascular, or digestive systems. Long-term drug use can lead to drug resistance, dependence, or withdrawal symptoms. Therefore, synthetic drugs, such as selegiline hydrochloride, paroxetine, dexamethasone, and ketamine, which are already in clinical use as antidepressants, have certain limitations [38]. For example, selegiline hydrochloride [39], a monoamine oxidase inhibitor, requires strict dietary control in order to prevent tyramine reactions. SSRIs, such as paroxetine [40] have a slow onset of action and often cause sexual dysfunction, insomnia, or weight gain. Long-term use of dexamethasone [41], a glucocorticoid, may cause metabolic disorders and immunosuppression. Although ketamine has a rapid onset of action, it has significant psychiatric side effects, such as hallucinations and dissociation, presents the risk of abuse, and has to be administered via intravenous infusion, which limits its widespread use. Therefore, rational formulation strategies to assist synthetic drugs to achieve potent antidepressant effects are crucial for clinical use. Currently, various types of NDDS are under preclinical investigation, including lipid-based, stimuli-responsive, gel-based, inorganic, and polymer-based nanoparticles (Table 3). Lipid-based nanoparticles include nanostructured lipid carriers (NLC), transferosomes, and nanoemulsions. Stimuli-responsive nanoparticles include those activated by near-infrared (NIR) light, magnetic fields, pH changes, or ROS. Gel-based nanoparticles are primarily nanogels, whereas inorganic nanoparticles often involve systems such as mesoporous silica nanoparticles. For synthetic drugs, especially those already approved for clinical use, nanomaterials are mainly employed to increase bioavailability, brain-targeting efficiency, and biodistribution, thereby improving antidepressant efficacy and reducing side effects. In the case of investigational drugs that are not yet commercially available, the research focus has shifted toward developing advanced formulations such as smart drug delivery systems capable of on-demand or site-specific drug release at targeted regions. Synthetic drugs are a core achievement of the modern pharmaceutical industry and provide a variety of effective tools for antidepressant treatment. However, inherent side effects and pharmacokinetic deficiencies limit achievement of their maximal clinical benefit. Improving the effectiveness of existing synthetic drugs through advanced drug formulation technologies and building more intelligent and precise drug delivery systems are important directions for overcoming these limitations and developing the next generation of antidepressant therapies, which are of key clinical significance in improving patients’ quality of life.

**Table 3:** Nano drug delivery system for synthetic drug delivery

Nano type	Main components	Synthetic drugs	Function	Antidepressant effects/mechanisms
NLC [39]	Gelatin, polyvinyl alcohol, polydimethylsiloxane	Selegiline hydrochloride	λ Stably controlled drug release λ Increased bioavailability	λ No pharmacodynamic evaluation
Nanotransferosomal gel [42]	Tween^®^80, PL 90 G	Paroxetine	Enhanced drug permeation and stability	λ Improved behavioral outcomes λ Increased neuronal survival rates λ Upregulated BDNF expression λ Reduced TNF-α levels in brain and plasma
Dual-responsive nanospheres [25]	Iron (III) chloride nonahydrate, trimesic acid, PVP	Domperidone	NIR and magnetic dual-responsive drug delivery	λ Improved the depressant-like behaviors λ Upregulated dopamine receptor density λ Repaired synaptic structure
Dual-responsive nanoformulations [43]	Lecithin, PEG2000, cyclodextrin	Dexamethasone	pH/ROS dual-responsive drug delivery	λ Decreased ROS and proinflammatory factors λ Relieved depressive symptoms
Nanogel [44]	Sodium hyaluronate	PACAP and estrogen	λ Penetrated to the BBB λ ROS-responsive drug delivery	λ Improved depressant-like behaviors λ Enhanced antioxidant and anti-inflammatory properties λ Promoted synaptic plasticity
Mesoporous silica nanoparticles [45]	Mesoporous silica, hyaluronic acid	Ketamine	λ Crossed the BBB λ Targeted to N-methyl-D-aspartate receptor (NMDAR)	λ Improved depressant-like behaviors and cognitive function λ Reversed deficits in synaptic proteins and plasticity
Nanoparticles [46]	Hexa-arginine, 4-hydroxymethylphenylboronic acid pinacol ester	Olanzapine and H_2_ donor amino borane	ROS-responsive drug delivery	λ Scavenged ROS and inhibited 5-HT dysfunction caused by oxidative stress λ Improved depressant-like behaviors
Nanoemulsion [47]	Chitosan, clove oil	Melatonin	Increased drug solubility and bioavailability	λ Improved depressant-like behaviors
NLC [48]	Compritol 888 ATO, Oleic acid, Tween 80	Levosulpiride	Increased bioavailability	λ Improved depressant-like behaviors λ Decreasing inflammatory levels
Nanoemulsion [40]	Caproyl 90, Cremophore EL, propylene glycol	Paroxetine	Increased bioavailability	λ Improved depressant-like behaviors
NLC [49]	Stearyl alcohol, oleic acid, Tween 80, Span 80 and Myrj 52	Agomelatine	Increased antidepressant activities	λ Improved depressant-like behaviors λ Improved neuronal morphology λ Reduced expression of inflammatory markers
Nanocomposites [50]	Chitosan, tocopherol polyethylene glycol 1000 succinate	Sulpiride	Increased bioavailability	λ Increased 5-HT and DA levels λ Attenuated oxidative stress state in brain
Polymer Nanoparticles [51]	Ni(cod)_2_, bipyridine, etc.	Fasudil	λ NIR-sensitive drug delivery λ Penetrated the BBB	No pharmacodynamic evaluation

### Gene and protein delivery

As a neurotrophic factor, brain-derived neurotrophic factor (BDNF) plays a crucial role in the regulation of depression. Activation of the BDNF–TrkB signaling pathway promotes neuronal survival, differentiation, and synaptic plasticity, thus contributing to the improvement of depressive symptoms. Therefore, exogenous supplementation with BDNF and activation of the BDNF‒TrkB pathway are considered potential antidepressant strategies [52]. However, there are several limitations in the in vivo delivery of BDNF. For example, when administered orally, BDNF is easily degraded in the gastrointestinal tract, resulting in an extremely low bioavailability. Furthermore, owing to its high molecular weight and hydrophilic nature, it cannot easily cross the BBB. To address this issue, Xu et al. developed a quercetin- and alginate-based nanogel delivery system to encapsulate BDNF [53]. This delivery system effectively crossed the BBB when administered via the intranasal route and achieved efficient brain delivery of BDNF [10]. Moreover, it utilized the antioxidant properties of quercetin to protect BDNF from oxidative damage, thereby providing a novel and efficient drug delivery strategy for the treatment of depression [54]. The results revealed that the NDDS exhibited significant antidepressant effects in both the reserpine-induced mouse model and chronic unpredictable mild stress (CUMS) rat model. The mechanism of action primarily involves the activation of the BDNF‒TrkB signaling pathway. In addition, NDDS represent a promising strategy for gene delivery. Researchers are attempting to address the limitations of current treatment methods, such as slow efficacy, poor patient tolerance, and numerous side effects, to effectively deliver the p11 gene, which affects serotonin regulation and function in the brain. Gandhi et al. synthesized two novel cationic lipids by combining the phospholipid 1,2-dioleoyl-sn-glycero-3-phosphoethanolamine (DOPE) with the essential amino acids histidine and arginine [55, 56]. These lipids were used to prepare liposomes and their performance as gene delivery vectors was evaluated. These liposomes were subsequently transformed into immunoliposomes using an IGF-II monoclonal antibody that targets the central nervous system [57]. The results showed that these liposomes possessed excellent targeting ability and transfection efficiency, suggesting their potential as novel gene delivery vectors for the treatment of depression. In summary, these NDDS provide innovative strategies for gene or protein delivery, overcoming their limitations in depression therapy.

### Dual antidepressant delivery

Single-component treatments have limited antidepressant effects. For example, the use of only certain antioxidants or neurotrophic factors often fails to comprehensively modulate multiple signaling pathways involved in depression [58, 59]. Therefore, dual- or multi-component strategies that synergistically modulate different targets through complementary pharmacological effects can achieve stronger and more comprehensive antidepressant effects [60]. Xu et al. prepared an alginate nanogel to co-deliver quercetin and BDNF for antidepressant therapy [53]. The antioxidant properties of quercetin help alleviate neuroinflammation and oxidative stress damage, thereby improving the neuronal microenvironment [61, 62]. BDNF promotes the activation of the BDNF‒TrkB signaling pathway, enhancing neurogenesis and synaptic plasticity [63]. Furthermore, the antioxidant activity of quercetin protects BDNF from oxidative degradation, thereby maintaining its bioactivity and further enhancing its antidepressant effects. Thus, the co-delivery of quercetin and BDNF via the alginate nanogel synergistically exerts neuroprotective and neurotrophic effects, improving the overall therapeutic outcome. Furthermore, the co-delivery of two active small-molecule compounds can increase overall antidepressant efficacy by synergistically modulating multiple pathways related to depression. Xu et al. developed an alginate-based nanogel that exhibited both brain targeting and a ROS response for the co-delivery of paeoniflorin and glycyrrhizinic acid to the brain [35]. The alginate nanogel not only efficiently crosses the BBB, but also possesses good biocompatibility and ROS-triggered drug release properties, enabling precise drug delivery in the oxidative stress microenvironment associated with depression. Paeoniflorin exerts its antidepressant effect by inhibiting neuroinflammation and modulating monoamine neurotransmitters, whereas glycyrrhizic acid has anti-inflammatory, antioxidant, and neuroprotective functions [64–66]. The synergistic effects of these compounds can simultaneously improve depressive-like behaviors and neuropathological states through multiple pathways. Therefore, this NDDS significantly enhances the antidepressant effects of paeoniflorin and glycyrrhizinic acid, resulting in a more comprehensive therapeutic effect. In summary, the NDDS can simultaneously deliver two antidepressants, thereby achieving a synergistic antidepressant effect. Leveraging the complementary mechanisms of these two antidepressants significantly enhances antidepressant efficacy, outperforming single-drug therapy.

### Current limitations and challenges

#### Drug delivery efficiency

Although nanocarriers (such as biomimetic nanoparticles and liposomes) can cross the BBB ​​and enhance the brain-targeting ability of drugs, their large-scale production presents significant challenges. For example, the heterogeneity of cell membrane–coated nanoparticles leads to inconsistent stability across batches, whereas the extraction efficiency of natural carriers, such as exosomes is low, and their purification process is complex [67]. Furthermore, certain NDDS, such as quercetin-loaded lipid nanoparticles, are prone to drug leakage during storage, and their release kinetics are difficult to precisely control, affecting the duration of therapeutic effects [68]. Targeted delivery efficiency is also limited by nanoparticle size; particles larger than 200 nm may be cleared by the mononuclear phagocyte system and surface charge, leading to uneven drug distribution in the brain. These issues decrease the efficiency of the delivery of antidepressants to the brain.

### Potential toxicity risks

Biocompatibility of nanomaterials remains a key issue. For example, iron-based nanoparticles may produce ROS via the Fenton reaction, causing oxidative stress damage to neurons, whereas the long-term use of polydopamine-based materials may induce neuroinflammation or mitochondrial dysfunction [69]. Furthermore, the long-term accumulation effects of nanoparticles are unclear. For example, abnormal deposition of silica nanoparticles in the olfactory bulb and hippocampus may disrupt neuronal signaling [70]. Some nanocarriers (such as cationic liposomes) may also disrupt the BBB, thereby exacerbating brain tissue damage. Therefore, the potential risks associated with nanomaterials are a limiting factor for their clinical application.

#### Insufficient mechanistic understanding

As nanoparticle formulations typically contain multiple materials and potentially therapeutic drugs, the regulatory mechanisms of these formulations are difficult to fully elucidate. For example, although alginate-based nanogels enhance the antidepressant effects of albiflorin, the regulatory mechanism of the nanogel itself remains unclear [71]. Furthermore, the protein corona formed by the interaction between the nanoparticle carrier and plasma proteins may alter its targeting properties; however, the underlying molecular mechanisms of this process have not been sufficiently investigated.

#### Barriers to clinical translation

There are significant differences between animal models and human pathologies. For example, LPS- or CUMS-induced depression models cannot accurately simulate the heterogeneity of chronic human stress, which limits the validation of the efficacy of nanomedicines. Furthermore, quality control standards for nanomedicines, such as particle size distribution and uniformity of surface modifications, have not yet been established, and there is a lack of standardized guidelines for translation from preclinical to clinical trials.

#### Sustainability issues

The preparation of certain nanomaterials involves complex chemical synthesis or bioengineering processes, such as precise control of size, morphology, and surface functionalization, resulting in high production costs and making large-scale production difficult under current conditions. Furthermore, the biocompatibility and long-term toxicity of many nanomaterials have not been systematically evaluated, and their potential for accumulation in the nervous system, immunogenicity, effects on metabolic pathways, and potential neuroinflammatory reactions remain unclear. Collectively, these factors limit the clinical translation and application prospects of nanomaterials in the treatment of depression.

## Conclusions

In this study, scientometrics was used to obtain an overview of nanomaterials for depression therapy. We analyzed and summarized the characteristics of publications; identified the distribution of research topics; identified the most influential countries, institutions, and journals; and identified research hotspots and trends in the field of nanomaterials for the diagnosis and treatment of depression. Based on the results of this scientometric analysis, we discussed the potential applications of nanomaterials for the diagnosis of depression, nano-bioactive materials for depression therapy, NDDS for antidepressant delivery, and the current limitations and challenges. Overall, nanomaterials hold significant promise for the development of novel diagnostic and therapeutic platforms for depression.

## Data Availability

Data sharing is not applicable to this article, as no datasets were generated or analyzed in the current study.
